# Immature CD4^+^CD8^+^ Thymocytes Are Preferentially Infected by Measles Virus in Human Thymic Organ Cultures

**DOI:** 10.1371/journal.pone.0045999

**Published:** 2012-09-24

**Authors:** Yukari Okamoto, Luca A. Vricella, William J. Moss, Diane E. Griffin

**Affiliations:** 1 W. Harry Feinstone Department of Molecular Microbiology and Immunology, Johns Hopkins Bloomberg School of Public Health, Baltimore, Maryland, United States of America; 2 Department of Epidemiology, Johns Hopkins Bloomberg School of Public Health, Baltimore, Maryland, United States of America; 3 Department of Cardiac Surgery, Johns Hopkins University School of Medicine, Baltimore, Maryland, United States of America; University of Pittsburgh, United States of America

## Abstract

Cells of the human immune system are important target cells for measles virus (MeV) infection and infection of these cells may contribute to the immunologic abnormalities and immune suppression that characterize measles. The thymus is the site for production of naïve T lymphocytes and is infected during measles. To determine which populations of thymocytes are susceptible to MeV infection and whether strains of MeV differ in their ability to infect thymocytes, we used *ex vivo* human thymus organ cultures to assess the relative susceptibility of different subpopulations of thymocytes to infection with wild type and vaccine strains of MeV. Thymocytes were susceptible to MeV infection with the most replication in immature CD4^+^CD8^+^ double positive cells. Susceptibility correlated with the level of expression of the MeV receptor CD150. Wild type strains of MeV infected thymocytes more efficiently than the Edmonston vaccine strain. Thymus cultures from children ≥3 years of age were less susceptible to MeV infection than cultures from children 5 to 15 months of age. Resistance in one 7 year-old child was associated with production of interferon-gamma suggesting that vaccination may result in MeV-specific memory T cells in the thymus. We conclude that immature thymocytes are susceptible to MeV infection and thymocyte infection may contribute to the immunologic abnormalities associated with measles.

## Introduction

Measles continues to be an important cause of child morbidity and mortality worldwide and many aspects of the pathogenesis of the disease remain poorly understood [1], [2]. Immune suppression accompanies infection and most measles deaths are due to infection with other pathogens [3]. Primary and secondary lymphoid tissues are important sites for measles virus (MeV) replication and B cells, T cells and monocytes are susceptible to infection [4]. Lymphopenia is characteristic of acute measles [5] and dysfunction of infected cells may contribute to immunologic abnormalities that include depressed delayed type hypersensitivity skin test responses [6]–[8], decreased mitogen-induced lymphoproliferation [9]–[11] and increased susceptibility to other infections [12], [13] and autoimmune disease [14]. Within these mononuclear cell populations, some subsets of cells are more susceptible to infection than others and this varies with virus strain [15], [16]. Identification of the cells of the immune system that are infected by wild type and vaccine strains of MeV is important for understanding the effects of MeV infection on the immune system.

One important determinant of cell tropism is the expression of cell surface molecules important for MeV entry. Three cellular receptors for MeV are recognized: the relatively low affinity complement regulatory protein CD46 [17], [18], present on all nucleated cells [19]; the higher affinity signaling lymphocyte activation molecule (SLAM/CD150) [20]–[22], present on subsets of activated lymphocytes and antigen-presenting cells [23]–[25]; and nectin-4 present on epithelial cells [26], [27]. These receptors interact primarily with the hemagglutinin (H) attachment protein on the surface of the virus, although virion-incorporated cellular proteins may also mediate entry into epithelial cells [28]. The H proteins of wild type (WT) strains of MeV preferentially bind CD150 [29]–[31], the primary determinant of MeV tropism for immune cells. Tissue culture-adapted and vaccine strains of MeV interact efficiently with CD46, as well as CD150 [32].

Lymphoid tissues, including the thymus, are major sites of WT MeV replication during natural infection of humans and experimental infection of nonhuman primates [33]–[36]. Because the thymus is the source of naïve T cells, thymic damage may contribute to prolonged immunologic abnormalities associated with measles [37]–[39]. The best characterized target cell in the thymus is the cortical stromal epithelial cell, which plays an important role in provision of the microenvironment necessary for differentiation of thymocytes and for generation and selection of the T cell repertoire [39]–[41]. *In vitro* infection of thymic epithelial cells with MeV induces terminal differentiation and apoptosis associated with production of type 1 interferon (IFN) [42], [43]. SCID-hu mice with co-implants of fetal human thymus and liver experimentally infected with tissue culture-adapted WT (Chicago-1) and WT (Bilthoven), but not vaccine (Moraten), strains of MeV show infection of thymic epithelial and myelomonocytic cells and rapid depletion of CD4^+^CD8^+^ double positive (DP) thymocytes by apoptosis [40].

Because MeV is a human virus, the systems available to study cell tropisms, strain differences and pathogenesis are limited. In general, these studies have focused on *in vitro* infection of human peripheral blood mononuclear cells or animal models, primarily nonhuman primates and transgenic mice. An intermediate approach is to use organ cultures of human tissue cultured *ex vivo*. For instance, studies of cultures of human tonsils have shown that B cells are particularly susceptible to infection [16], [44]. Within T cell populations, WT viruses are more likely to infect CD150-expressing memory T cells than CD150-negative naïve T cells while vaccine strains also infect naïve T cells, presumably because they can use CD46 as an additional receptor [15], [16]. To better understand the susceptibility of immature T cells to infection and the potential role of the thymus in measles pathogenesis, we studied MeV infection in organ cultures of human thymus.

**Figure 1:**
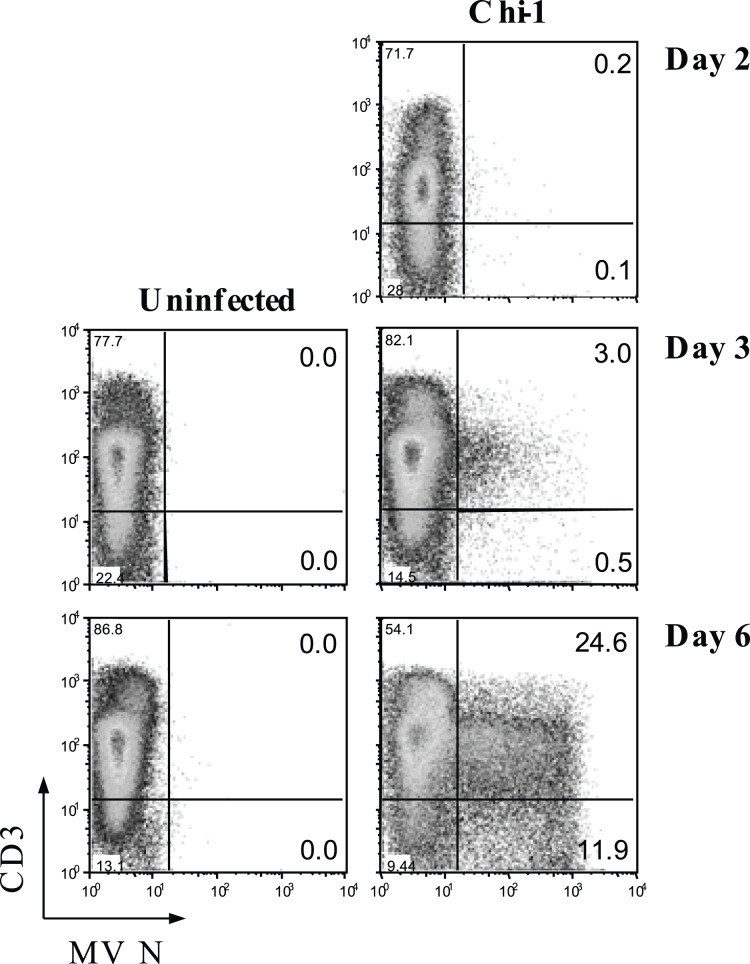
MeV infection of thymocytes in TOC. TOCs from a 1 year-old child were inoculated with Chi-1 or control media at 2×10^−4^ pfu/cell and the MeV N protein expression in thymocytes was monitored for 6 days. Thymocytes were double-stained for N protein and cell surface CD3 and analyzed by flow cytometry. These data are representative of TOCs from 3 different donors.

## Materials and Methods

### Ethics Statement

Informed written consent for cardiac surgery and research use of discarded tissue was obtained from the parents or guardians of the children in this study. The protocol and consent procedures were approved by the Institutional Review Board of the Johns Hopkins Bloomberg School of Public Health.

**Figure 2:**
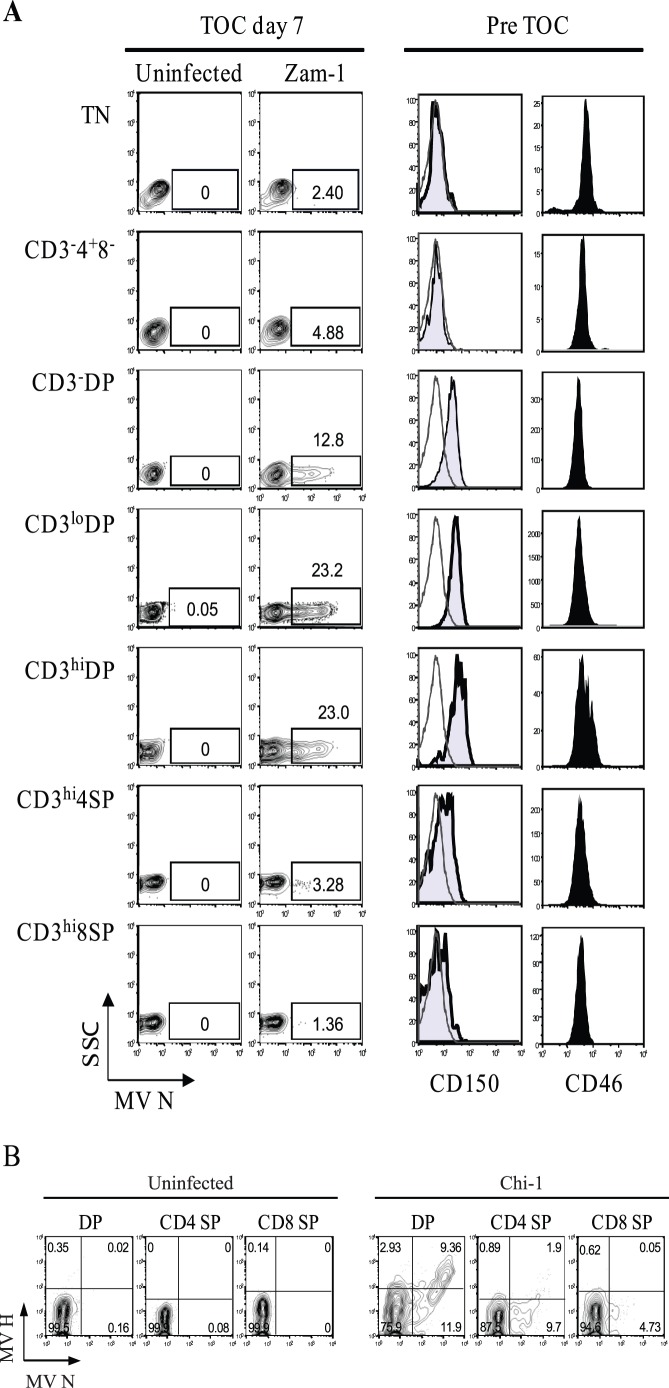
Preferential infection of MeV in CD4^+^CD8^+^ double-positive thymocytes in TOC. (**A**) TOCs from a 6 month-old child were inoculated with Zam-1 at 2×10^−4^ pfu/cell or control media. Thymocytes were stained for surface markers CD3, CD4, CD8, and intracellular MeV N protein on Day 7. N protein expression is shown for thymocyte subsets classified according to the expression of surface markers. The numbers in the dot plots (left two columns) indicate the percentage of N protein-positive cells (squares). TN cells are immature thymocytes negative for CD3, CD4 and CD8 but this population will also contain B, NK, and γδ-T cells. Some thymocytes were set aside before TOC for CD150 and CD46 staining (right two columns). These data are representative of TOCs from 5 different different donors. (**B**) TOCs from a 3 day-old child were inoculated with Chi-1 or control media at 2×10^−4^ pfu/cell and thymocytes were stained for CD4, CD8, N protein and cell surface H protein at day 7. These data are representative of TOCs from 4 different donors.

### Viruses

Three strains of MeV were studied: a vaccine strain, Edmonston B (Edm B, genotype A; American Type Culture Collection), a tissue culture-adapted WT strain, Chicago-1 (Chi-1, genotype D3) [45] and a non-adapted WT strain, Zambia-1 (Zam-1, genotype D2) [46]. Edm B and Chi-1 were grown and assayed by plaque formation in Vero cells that do not express CD150. Zam-1 (E996) was isolated in B95-8 cells [37] and then grown and assayed in Vero cells stably expressing human CD150 [31]. For stocks, culture supernatant fluids were centrifuged at 3,000 rpm for 10 min, dialyzed against PBS using a 300,000 MW cut-off membrane (Millipore), disbursed in aliquots and stored at –80°C. All virus stocks and cell lines were negative for mycoplasma (MycoAlert Kit, Lonza).

**Figure 3:**
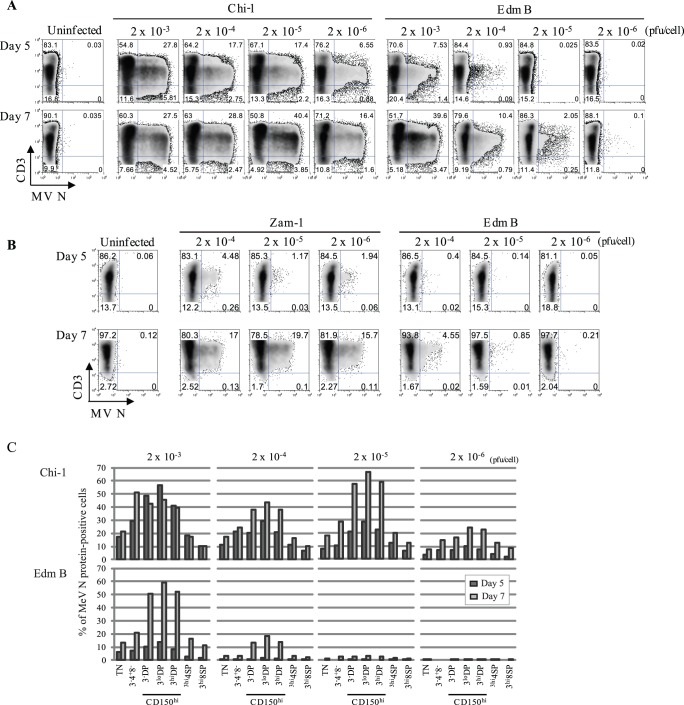
Differential susceptibility of thymocytes to infection with vaccine and WT MeV strains. (**A**) TOCs from an 8 month-old child were inoculated with Chi-1 or Edm B in amounts ranging from 2×10^−3^ to 2×10^−6^ pfu/cell. Thymocytes were examined for MeV N protein on days 5 and 7 after infection. These data are representative of TOCs from 4 different donors. (**B**) TOCs from an 11 month-old child were inoculated with Zam-1 or Edm B in amounts ranging from 2×10^−4^ to 2×10^−6^ pfu/cell. Thymocytes were examined for MeV N protein on days 5, 7 and 9 after infection. These data are representative of TOCs from 3 different donors. (**C**) Thymocytes from the experiment described in (A) were analyzed for surface CD3, CD4, and CD8, as well as MeV N protein expression. Percentages of cells infected (positive for N protein) for each surface phenotype are shown (upper panels: Chi-1, lower panels: Edm B).

### Thymus Organ Culture and Virus Infection

Samples of thymus tissues were obtained from 17 children aged 3 days to 7 years undergoing corrective cardiac surgery at the Johns Hopkins Hospital and were processed within 6 h. Thymus organ cultures (TOCs) were established as previously described [47]. Briefly, thymus tissue was dissected into pieces of approximately 2 mm^3^ so that every piece would contain cortex and medulla with similar cellularity, and each contained ∼3×10^6^ cells. Fragments were cultured on polyethylene terephthalate track-etched trans-well membranes (Becton Dickinson) suspended in 12-well or 6-well plates in UltraCULTURE fetal bovine serum (FBS)-free medium (Lonza) supplemented with 50 U/mL penicillin and 50 µ g/mL streptomycin. Infections used 2×10^−3^, 2×10^−4^, 2×10^−5^ or 2×10^−6^ pfu/cell and cultures were maintained in a 5% CO_2_ incubator at 37°C with daily replacement of medium.

**Figure 4:**
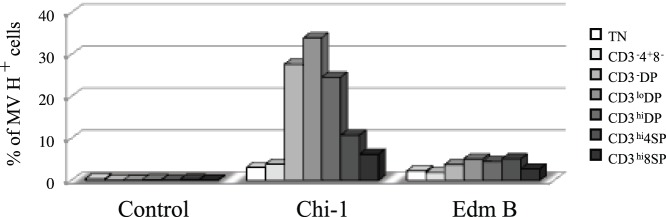
Differential binding of MeV strains to subpopulations of thymocytes. Chi-1, Edm B, or control supernatant fluid was incubated with thymocytes from a 5 year-old girl at 4°C and cell surface-associated MeV H protein and CD3, CD4 and CD8 were detected by flow cytometry. Bars show the percentage of H protein-positive cells in each population of thymocytes.

### Flow Cytometric Analysis

For staining of each surface marker, three thymic fragments were combined. For isolation of thymocytes, fragments were mechanically disrupted or digested with collagenase D (0.5 mg/ml), collagenase/dispase (1 mg/ml) and DNase (0.05 U/ml) (Roche Diagnostics) for 2 hours at 37°C in 5% CO_2_. Isolated thymocytes were incubated in the staining buffer (Hank’s balanced salt solution supplemented with 1% BSA) with 20% human AB serum (Mediatech) prior to staining. The following monoclonal antibodies and isotype-matched control antibodies were used for staining: FITC-labeled anti-MeV nucleoprotein (N) (83 KKII, Chemicon), anti-MeV H (CV4 blend, Chemicon), fluorescently labeled-anti-CD3, CD4, CD8, CD46, and CD150 (BD Pharmingen or BD Bioscience). For combined staining, cells were first stained with anti-MeV-H followed by APC-labeled goat anti-mouse IgG, washed and then stained for other surface markers. For intracellular MeV N protein staining, the cells were first stained for surface antigens and then fixed and permeabilized using the Cytofix/Cytoperm kit (BD Pharmingen) prior to staining for MeV N. After staining, cells were fixed in 1% paraformaldehyde and 4-color flow cytometry was performed using a FACSCalibur (Becton Dickinson). The data were analyzed using FlowJo software (Tree-Star, version 8.8.6).

**Figure 5:**
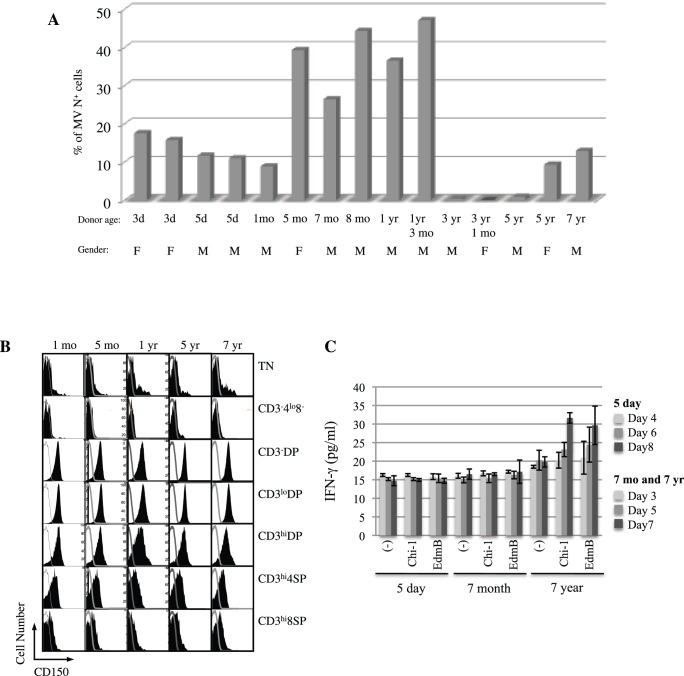
MeV susceptibility of thymus samples from children of different ages. (**A**) The maximum percentage of N protein-positive thymocytes for Chi-1-infected TOCs from 15 children. The peak of MeV replication was observed at day 6, 7 or 8 after infection depending on the TOC and the multiplicity of infection (2×10^−4^, 2×10^−5^ or 2×10^−6^). (**B**) Expression of CD150 was examined and compared between five uninfected thymuses from donors of different ages (1 and 5 months and 1, 5 and 7 years) that are also represented in (A). CD150 expression is shown in populations classified by CD3, CD4, and CD8 expression levels. (**C**) IFN-gamma was measured by EIA (triplicate wells) in the culture supernatant fluid at days 3, 5 and 7 of TOCs from donors of ages 7 months and 7 years, or at days 4, 6 and 8 of a TOC from a 5 day-old donor, inoculated with Chi-1, Edm B or control.

### MeV Binding Assay

Single thymocytes isolated by mechanical disruption were washed with Hank’s balanced salt solution, and 3×10^4^ cells/well were incubated at 4°C for 4 hours with MeV (Chi-1 or Edm B) at 0.01 pfu/cell in 100 µ l RPMI 1640. Cells were washed 4 times with staining buffer and cell-associated MeV was detected by cell surface staining with anti-MeV H and APC-conjugated goat anti-mouse IgG antibodies. Cells were analyzed by flow cytometry.

### Interferon Assays

Cultures with 5 thymic fragments/well in a 12-well plate were inoculated in triplicate with MeV (Chi-1 or Edm B) at 2×10^−4^ pfu/cell. The supernatant fluids were recovered from MeV-inoculated or control uninfected cultures at 4, 6 and 8 days (5 day-old child) or 3, 5 and 7 days (7 month- and 7 year-old children). Levels of IFN-alpha (Biomedical Lab) and IFN-gamma (R&D Systems) were measured by enzyme immunoassay (EIA) according to the manufacturer’s instructions.

## Results

### MeV Infection of Thymocytes in TOC

To determine the susceptibility of thymocytes to MeV infection, TOCs were inoculated with Chi-1 or control culture supernatant fluid, and thymocytes were monitored for 6 days for expression of intracellular MeV N protein by flow cytometry (Fig. 1). N protein was detectable by 2 days after virus inoculation and by day 6 approximately 36% of thymocytes were positive for N protein.

### Preferential Infection of CD4^+^CD8^+^ Double-positive Thymocytes

T cell maturation in the thymus progresses from CD3^−^ to CD3^+^ and from early CD4^+^ to immature CD4^+^CD8^+^ DP before acquiring the mature CD3^+^CD4^+^ and CD3^+^CD8^+^ single positive (SP) phenotypes [39]. To determine the thymocyte populations susceptible to WT MeV, we examined MeV N protein expression in individual populations representing different thymic maturation stages 7 days after infection with Zam-1 (Fig. 2A, left two columns). Immature CD4^+^CD8^+^ DP thymocytes were most susceptible to infection, with about 12.8% of CD3^−^ DP, 23.2% of CD3^lo^ DP and 23% of CD3^hi^ DP cells positive for N protein compared to 3.28% of CD4^+^ SP or 1.36% of CD8^+^ SP cells.

To determine whether expression of the CD46 or CD150 MeV receptors accounts for the preferential infection of DP thymocytes, receptor surface expression was examined before culture (Fig. 2A, right two columns). Expression of CD150 was highest on immature DP thymocytes, while expression of CD46 showed little difference between thymocyte populations.

To determine whether the presence of MeV N protein indicated active viral replication, we evaluated expression of MeV H on the surface of infected thymocytes (Fig. 2B). Most thymocytes strongly positive for N protein also expressed H protein indicating that human thymocytes support productive replication of MeV.

### Comparative Susceptibility of Thymocytes to a Vaccine and a Laboratory-adapted WT Strain of MeV

To determine whether there is any difference between vaccine and laboratory-adapted WT MeV strains in infectivity for thymocytes, we infected TOC using varying amounts of the Edm B vaccine virus and Chi-1, a clinical isolate that replicates in CD150-negative Vero cells [48] and assessed efficiency of infection (Fig. 3). Both strains can use CD46 as well as CD150 as receptors, but differ in virulence for monkeys [49]. TOCs were inoculated at multiplicities of 2×10^−3^ to 2×10^−6^ pfu/cell and thymocytes were stained for N protein at day 5 and day 7 (Fig. 3A). Chi-1 infected thymocytes more efficiently than Edm B. At day 5 Chi-1-infected thymocytes were detected even at the lowest multiplicity (2×10^−6^ pfu/cell) with about 7% N protein-positive cells, similar to that obtained with Edm B at the higher moi of 2×10^−3^ pfu/cell. This indicates a difference in infectivity of approximately 1000-fold. The higher level of replication of Chi-1 at day 7 at a lower moi is consistent with observations of MeV replication in other cell types where formation of defective interfering RNAs at a high moi inhibits virus replication [50]. Alternatively, failure of infected cells to increase after infection with Chi-1 at a multiplicity of 2×10^−3^ may reflect loss of DP cells due to apoptosis [40], [47]. Although Edm B inoculation at 2×10^−3^ pfu/cell resulted in a substantial number (∼44%) of N protein-positive cells by day 7, the level of N protein expression indicated a lower level of replication compared to Chi-1. Similar data were obtained when infection with the WT non-adapted Zam-1 strain was compared with the Edm B vaccine strain (Fig. 3B). Analyses of the populations of thymocytes infected (Fig. 3C) indicated that Chi-1 infected all populations more efficiently than Edm B and both strains replicated most efficiently in immature DP thymocytes.

To determine whether differences in infectivity derived from differential abilities of the two strains to bind to thymocytes, we incubated thymocytes with each virus at 4°C and assessed virus bound to the cell surface using antibody to the H protein (Fig. 4). Chi-1 bound to all thymocyte subsets more efficiently than Edm B. Immature DP cells, which have the highest expression of CD150 (Fig. 2A), showed the highest binding of Chi-1. Of note, Edm B showed little variation in binding to the different subsets, perhaps due to use of CD46 that is similarly expressed by all thymocyte subsets (Fig. 2A). Nevertheless, immature DP cells were more likely to become infected with Edm B than with the other strains (Fig. 3C).

### MeV Susceptibility of Thymus Samples from Subjects of Different Ages

In the course of these studies using TOC, we identified cases where the explant cultures did not support MeV infection. To identify the correlates of successful infection we compared the susceptibility of 15 different thymuses to MeV infection using Chi-1 (Fig. 5A). In general, thymuses from very young infants (≤1 month) or older children (≥3 years) were less susceptible to Chi-1, while the intermediate-aged thymuses readily supported Chi-1 replication. The sex of the donors did not appear to affect susceptibility. Comparison of surface CD150 expression in several thymus samples did not reveal a difference between thymuses from children of different ages (1 and 5 months and 1, 5 and 7 years) (Fig. 5B). The relative proportions of immature and mature thymocytes were not different between these thymuses (data not shown).

Because type I IFN and IFN-gamma play major roles in innate and adaptive antiviral defenses, and are capable of suppressing MeV replication in some cells [51]–[54], we examined IFN production by TOC from different ages in response to MeV exposure (Fig. 5C). IFN-gamma production was increased after exposure to both Chi-1 and Edm B in the TOC from a 7 year-old child, while there was no induction of IFN-gamma in the TOC from 5 day-old and 7 month-old children. IFN-alpha levels were below the limit of detection in all samples (data not shown).

## Discussion

These studies show that thymocytes are susceptible to infection with all strains of MeV tested. Immature CD4^+^CD8^+^ DP cells were most susceptible and susceptibility correlated with high expression of CD150 by this population of cells. DP thymocytes were more efficiently infected with WT strains of MeV than a vaccine strain. Thymus organ cultures from older children were often resistant to MeV infection and in one child this was associated with production of IFN-gamma within the culture.

The thymus is a target organ for MeV infection [55], but the cells infected by MeV have not been well characterized. Thymic epithelial cells are susceptible to MeV infection *in vitro* [42], [43] and in human thymus/liver implants in SCID-hu mice [40]. Human autopsy studies have shown immunocytochemical staining for MeV in Hassell’s corpuscles formed from epithelial cells in the medulla [34] and in Warthin-Finkeldey giant cells formed from T cells in the cortex [36], suggesting that both populations may be involved in natural infection. Mice transgenic for human MeV receptors have provided a mixed picture of thymus cell susceptibility. Transgenic mice expressing human CD46 show virus replication in the thymus primarily in macrophages and dendritic cells [56], while MeV infection in the thymus of newborn transgenic mice expressing human CD150 is found in DP and CD4^−^CD8^−^ double negative cells [57]. Stat-1−/− transgenic mice expressing the human CD150 gene also showed MeV RNA in the thymus [58], while IFNAR−/− mice with a knock-in chimeric human CD150 gene showed no infection in the thymus [59].

Our results demonstrate the susceptibility of DP human thymocytes to infection. DP cells are found primarily in the thymic cortex where they interact with morphogen-producing epithelial cells [41] that are also susceptible to infection [40]. The studies further suggest that CD150 plays an important role in MeV infection of DP thymocytes. This is consistent with the known distribution of human CD150 on DP thymocytes, as well as activated B and T lymphocytes and monocytes [25], [60]. However, receptor binding may not be the only factor dictating susceptibility to infection. CD4^+^ SP cells bound Edm B at comparable levels to immature DP cells, but were substantially less likely to express viral proteins. Because immature DP thymocytes are the most actively proliferating cells in TOC [47] and MeV replicates more efficiently in mitogen-stimulated lymphocytes [61], proliferation status of DP cells may also enhance viral replication.

Some TOCs were resistant to MeV infection suggesting inhibition of virus replication that was correlated with the age of the child. We do not have measles vaccination histories for these children but presume that children over the age of 2 years would have received the live virus vaccine sometime between 12 and 15 months of age and be MeV-immune. Although the thymus is a primary lymphoid organ, antigen-specific memory T cells can re-enter the thymus where they are detected in the medulla [62]. Lack of MeV replication after infection of TOC from older children may reflect the presence and activation of MeV-specific memory T cells resulting in suppression of MeV replication. IFN-gamma, detected in the culture of one older child, exerts an antiviral effect on epithelial, endothelial and astroglial cell lines, but not in B lymphoblastoid cell lines through induction of indoleamine 2,3-dioxygenase and perhaps other mediators that protect against MeV infection [51]. TOCs from very young infants (<1 month) were also less susceptible than TOCs from children between 5 and 15 months, possibly due to high levels of residual maternal antibody [63]. Identification of the actual mechanism of age-dependent protection from MeV infection in TOCs will require further investigation of larger numbers of children.

The importance of thymus infection for MeV-induced immunosuppression and predisposition to autoimmune disease is unclear. During measles there is lymphopenia, but this is transient and changes in the proportions of CD4^+^ and CD8^+^ T cells in circulation are modest [5]. Maintenance of naïve T lymphocytes in circulation is dependent on continued production by the thymus. Changes in production of naïve T cells can be measured by quantifying T cell receptor rearrangement excision circles in the T cell population. During measles there is no deficit in production of CD4^+^ or CD8^+^ SP thymic emigrants, suggesting that a decrease in thymic output is not the cause of lymphopenia or depressed cellular immunity [64]. However, infection may allow thymocyte release prior to negative selection [39] and have long-term effects on the T cell repertoire through depletion of immature DP thymocytes.
